# Graft weight integration in the early allograft dysfunction formula improves the prediction of early graft loss after liver transplantation

**DOI:** 10.1007/s13304-022-01270-0

**Published:** 2022-03-19

**Authors:** Tommaso Maria Manzia, Quirino Lai, Hermien Hartog, Virginia Aijtink, Marco Pellicciaro, Roberta Angelico, Carlo Gazia, Wojciech G. Polak, Massimo Rossi, Giuseppe Tisone

**Affiliations:** 1 Department of Surgery Science, University of Rome Tor Vergata, U.O.C. Chirurgia Epatobiliare e Trapianti, Fondazione PTV, Rome, Italy; 2 Department of Surgery and Organ Transplantation, Sapienza University of Rome, Rome, Italy; 3 Division of Hepatopancreatobiliary and Transplant Surgery, Department of Surgery, Erasmus MC, University Medical Center Rotterdam, Rotterdam, The Netherlands; 4 General Surgery and Organ Transplantation Unit, Department of General and Specialistic Surgery, Umberto I Polyclinic of Rome, Sapienza University of Rome, Viale del Policlinico 155, 00161 Rome, Italy

**Keywords:** Liver transplantation, Early allograft dysfunction, Graft weight, Graft loss

## Abstract

The role of the graft-to-recipient weight ratio (GRWR) in adult liver transplantation (LT) has been poorly investigated so far. The aim is to evaluate the contribution of the GRWR to the well-recognized early allograft dysfunction (EAD) model (i.e., Olthoff model) for the prediction of 90-day graft loss after LT in adults. Three hundred thirty-one consecutive adult patients undergoing LT between 2009 and 2018 at Tor Vergata and Sapienza University in Rome, Italy, served as the Training-Set. The Validation-Set included 123 LTs performed at the Erasmus Medical Center, Rotterdam, the Netherlands. The mEAD model for 90-day graft loss included the following variables: GRWR $\le$ 1.57 = 2.5, GRWR $\ge$ 2.13 = 2.5, total bilirubin ≥ 10.0 mg/dL = 2.0, INR ≥ 1.60 = 2.3, and aminotransferase > 2000 IU/L = 2.2. The mEAD model showed an AUC = 0.74 (95%CI = 0.66–0.82; *p* < 0.001) and AUC = 0.68 (95%CI = 0.58–0.88; *p* = 0.01) in the Training-Set and Validation-Set, respectively, outperforming conventional EAD in both cohorts (Training-Set: AUC = 0.64, 95%CI = 0.57–0.72; *p* = 0.001; Validation-Set: AUC = 0.52, 95%CI = 0.35–0.69, *p* = 0.87). Incorporation of graft weight in a composite multivariate model allowed for better prediction of patients who presented an aminotransferase peak > 2000 IU/L after LT (OR = 2.39, 95%CI = 1.47–3.93, *p* = 0.0005). The GRWR is important in determining early graft loss after adult LT, and the mEAD model is a useful predictive tool in this perspective, which may assist in improving the graft allocation process.

## Introduction

Liver transplantation (LT) is the only cure for many acute and chronic end-stage liver diseases and selected liver tumors. Early allograft dysfunction (EAD) is a commonly used tool for evaluating the initial graft function after LT. The most cited and internationally accepted definition of EAD, as proposed by Olthoff et al. is based on the presence of one or more the following factors: an increase in aspartate aminotransferase (AST) or alanine aminotransferase (ALT) above 2000 IU/L within the first week after LT, serum bilirubin ≥ 10 mg/dL and/or an INR ≥ 1.6 on day seven after LT [1]. It is well accepted a significant association between EAD and graft loss [1–6]. The role of the graft-to-recipient weight ratio (GRWR) on graft loss in deceased donor LT has been poorly investigated so far. The rationale for this study was based on the following hypotheses: (i) heavy livers are more challenging to perfuse at the time of multi-organ procurement; (ii) at the time of implantation and revascularization, the perfusion of larger livers might be slower and more complex since the blood flow must occupy a wider surface area and (iii) high GRWR can influence the graft outcome because of technical difficulties at implantation and compression of nearby organs (i.e., inferior vena cava and diaphragm). Accordingly, the primary aim of the present study was to explore whether a modified EAD model (mEAD) [1], including the GRWR, could improve the prediction of 90-day graft survival. The secondary aim was to evaluate the correlation between graft weight (GW) and the post-LT transaminase peak (T-peak).

## Methods

We retrospectively analyzed the data collected prospectively from three European liver transplant centers. Three hundred thirty-one consecutive patients who underwent LT between January 2009 and December 2018 in two inter-consortium Roman transplant centers (Tor Vergata and Sapienza University of Rome) were analyzed to produce the model derivation cohort (Training-Set). One hundred twenty-three patients from the Liver Transplant Unit of the Erasmus MC, University Medical Center, Rotterdam, The Netherlands, who underwent LT between July 2011 and October 2016, were used to validate the mEAD (Validation-Set). The exclusion criteria were patients who received livers from donors after cardiac death; patients subjected to a multi-organ transplant, split liver transplantation, or re-transplantation; patients who received livers after the machine perfusions; living donor liver transplantation and pediatric recipients.

The manuscript was approved by the local Ethical Committee of the Fondazione Policlinico Tor Vergata (reference 253/19).

### Organ procurement and liver transplantation

The grafts were perfused with Celsior solution during organ procurement, being procured during the “cold phase” within 30 min [7]. The organs were then cold-stored at 4 °C [8]. Baseline liver biopsies were always collected during organ procurement or at the backbench as a local practice. In the Training-Set cohort, livers were weighed using a digital scale after removing the surrounding non-hepatic tissues. Since the GW in the Validation-Set were not available at the time of data collection, after a careful evaluation of the available formulas in the English literature and an estimation of the acceptable discrepancy between the actual and estimated weight in the Training-Set [9] the weight was estimated (estGW) using the following formula: estimated standard liver weight (g) = 218 + body weight (kg) × 12.3 + gender (female 0; male: 1) × 51] [10]. In the Training and Validation-Set, the liver biopsies were analyzed by the same local expert pathologists at Tor Vergata University, Sapienza University, and Erasmus MC, respectively. Macrovesicular steatosis was classified as absent, mild (5–30%), and moderate (30–60%). Livers with steatosis ≥ 40% were considered for LT only after hypo- or normothermic machine perfusion assessment; therefore, they were not considered in the current analysis.

LTs were performed with the modified piggyback technique [11, 12]. The location of arterial anastomosis was considered case by case following the quality of donor and recipient arteries and vascular anatomy. After graft implantation, all patients underwent intraoperative Doppler ultrasonography to check the vascular patency before and immediately after closure.

### Demographics of donors and recipients

#### Training-set

Recipients’ data included age at LT, biochemical MELD score at the listing, indication for LT, ICU stay (days), duration of LT (minutes), total bilirubin (mg/dL), INR, AST (IU/L), ALT (IU/L) and incidence of EAD and graft loss. Donors’ data included: cause of death (trauma or other), age, gender, height, weight, body mass index (BMI), severe hypotension, regional share, warm and cold ischemic time, body surface area (BSA), liver weight and GRWR, graft loss (within 90 days) and mortality (within 90 days), biochemical parameters (AST, bilirubin, and sodium), biopsy-proven macrovesicular steatosis and EAD [2].

#### Validation-set

Recipients’ data included age at LT, biochemical MELD score at the listing, indication for LT, ICU stay (days), duration of LT (minutes), total bilirubin (mg/dL), post-liver transplant INR, AST (IU/L), ALT (IU/L), graft loss (within 90 days) and mortality (within 90 days). Donors’ data included age, gender, height, weight, BSA, warm and cold ischemic time, biopsy-proven macrovesicular steatosis, and EAD.

According to the primary and secondary end-points, the study follow-up was cut at 3 months. Serum aminotransferases were analyzed daily within the first postoperative week. The highest level of AST or ALT was considered the T-peak. Graft loss was defined as patient death or re-transplantation within the first 90 postoperative days.

### Statistical analysis

Continuous variables were reported as medians and interquartile ranges (IQRs); the Mann–Whitney *U* test was used to compare groups. Categorical variables were reported as numbers and percentages; comparisons were performed using Fisher’s exact test.

In the absence of internationally well-recognized threshold values, we identified two different GRWR cut-off values in the Training-Set able to divide the whole cohort into three groups: low GRWR (< 1.57, *n* = 81); intermediate GRWR (1.57–2.13; *n* = 167); and high GRWR (> 2.13, *n* = 83). The thresholds corresponded to the first quartile (1.57) and the third quartile (2.13).

Due to the sample size (*n* = 331) of the Training-Set and the end-points of the study, the univariate selection data were not computed as suggested by Heinze et al. [13]. The preoperative variables capable of predicting the 90-day risk of graft loss were analyzed using multivariate logistic regression (backward conditional). Odds ratios (ORs) and 95% confidence intervals (95% CIs) were computed for significant variables. Receiver-operating-characteristic (ROC) analysis was used to validate the model’s predictive ability in the Validation-Set cohort, and the area under the curve (AUC) and 95% CI were reported. The added value of incorporating the GRWR into the EAD model was reported through the category-based net reclassification improvement (NRI) [14]. The AUCs were compared using the z-test proposed by DeLong et al. [15]. The relative risks for 90-day graft loss were calculated for different classes of GRWR and mEAD. We used the median values of GRWR (value = 1.9–2.0) and mEAD (value = 4–5) as the referral values. The relative risks were calculated using the online calculator at https://www.omnicalculator.com/statistics/relative-risk.

The 90-day patient and graft survival probabilities were estimated using the Kaplan–Meier method. Differences were considered statistically significant at a *p* value < 0.05. All data were initially recorded within an EXCEL database (Microsoft, Redmond, Washington, United States) and then analyzed using the SPSS statistical package version 23.0 (SPSS Inc., Chicago, IL).

## Results

### Training-set

In the Training-Set cohort (*n* = 331), 245 patients (74%) were males, the median age at LT was 57 years (IQR: 50–62), and the biochemical MELD score was 15 (IQR:11–20).

A total of 203 (61.3%) patients experienced EAD after LT; of these, 28 (13.8%) simultaneously presented with all three EAD features, while 97 (47.8%) presented with only one feature, namely INR ≥ 1.6 (*n* = 46), T-peak > 2000 IU/L (*n* = 7), bilirubin ≥ 10 mg/dL (*n* = 44), and 78 (38.4%) with two features, namely aminotransferase and bilirubin (*n* = 7), aminotransferase and INR (*n* = 21), and bilirubin and INR (*n* = 50). Fifty-three (16%) patients experienced graft loss during the follow-up.

After LT, the median AST and ALT peaks were 927 (IQR: 530–1830) IU/L and 673 (IQR: 469–1270) IU/L, respectively. The median INR and serum bilirubin on day 7 were 1.5 (IQR: 1.26–1.96) and 8.8 (IQR: 5.2–14) mg/dL, respectively.

The median GW and GRWR were 1350 g (IQR: 1191–1648) and 1.83 (IQR: 1.56–2.13), respectively. Eighty-one (24.4%) recipients showed a GRWR < 1.57, 167 (50.4%) between 1.57 and 2.13, and 83 (25.1%) > 2.13. The abdomen was primarily closed in all patients.

The 90-day patient and graft survival rates were 85% and 84%, respectively. Five (1.5%) patients underwent re-transplantation due to primary non function (*n* = 4) or hepatic artery thrombosis (*n* = 1). Two (0.6%) patients died after re-transplantation, 24 due to infection/sepsis (7.1%), 20 (6.0%) for multiorgan failure and 7 (2.1%) due to other causes. The patients’ characteristics are summarized in Table 1.

**Table 1:** Main clinical characteristics of the Training-SET group according to GRWR (*n*:331)

Variables	GRWR < 1.57 (*n*:81) (% or IQR)	GRWR = 1.57–2.13 (*n*:167)	GRWR > 2.13 (*n*:83)	*p* value
MELD	15 (11–21.5)	15 (11–20)	15 (11–21)	0.889
Recipient age	57 (50–61.5)	58 (51–63)	56 (48–62)	0.460
Cirrhosis (*n* = 175, 52.9%)	46 (26.3)	83 (47.4)	46 (26.3)	0.499
•HCV (*n* = 45, 25.7%) •HBV (*n* = 21, 12%) •Alcohol (*n* = 48, 27.4%) •Cholestatic (*n* = 6, 3.4%) •Other (*n* = 47, 26.9%) •Unknown (*n* = 8, 4.6%)	13 (28.9) 7 (33.3) 15 (31.2) 1 (16.7) 9 (19.1) 1 (12.5)	22 (48.9) 11 (52.4) 20 (41.7) 4 (66.7) 22 (46.8) 4 (50)	10 (22.2) 3 (14.3) 13 (28.9) 1 (16.7) 16 (34) 3 (37.5)	
HCC (*n* = 156, 47.1%)	35 (22.4)	84 (53.8)	37 (23.7)	0.271
•HCV (*n* = 71, 45.5%) •HBV (*n* = 26,16.7%) •Alcohol (*n* = 31, 19.9%) •Cholestatic (*n* = 1, 0.6%) •Other (*n* = 20, 12.8%) •Unknown (*n*= 7, 4.5%)	18 (25.4) 8 (30.8) 5 (16.1) 0 3 (15) 1 (14.3)	32 (45.1) 12 (46.2) 22 (71) 1 (100) 11 (55) 6 (85.7)	21 (29.6) 6 (23.1) 4 (12.9) 0 6 (30) 0	
Donor age	58 (42–68)	61 (43–71)	60 (40–69)	0.835
Donor gender (M)	26 (32.1)	94 (56.3)	62 (74.7%)	< 0.001
Donor height (cm)	165 (160–170)	170 (162–175)	174 (168–180)	< 0.001
Donor weight (Kg)	67 (60–77.5)	74 (70–80)	80 (75–85)	< 0.001
Donor AST (IU/L)	45 (28–74)	33 (21–66)	42 (21–74)	0.070
Donor Na (peak) (mmol/L)	151 (145–158)	152 (146–157)	151 (147–155)	0.383
Donor severe hypotension or cardiac arrest	21 (25.9)	42 (25.1)	21 (25.3)	1.000
Donor BMI	24.7 (22–26.1)	26 (23.9–27.8)	26.5 (24.7–29.4)	< 0.001
Trauma	24 (29.6)	36 (21.6)	22 (26.5)	0.873
Donor ICU stay (days)	4 (2–6)	4 (2–6)	4 (3–7)	0.579
Regional Share (yes)	30 (37)	58 (34.7)	42 (50.6)	0.047
MacroSteatosis > 30%	6 (6)	10 (7.4)	7 (8.4)	0.781
WIT (min)	52 (47–59.5)	50 (42–56)	52 (45–55)	0.236
LT time (min)	428 (380–455)	430 (395–470)	430 (390–440)	0.238

*AST* aspartate aminotransferase, *Na* sodium, *BMI* body mass index, *HCC* hepatocellular carcinoma, *WIT* warm ischemic time, *ICU* intensive care unit, *LT* liver transplantation

### Validation-set

In the Validation-Set (*n* = 123), 62 recipients (50, 4%) were male; the median age at LT was 55 years (IQR: 44–61), and the biochemical MELD score was 15 (IQR: 11–21). The indications for LT were cirrhosis in 77 (62.6%) recipients and hepatocellular carcinoma in 45 (36.5%). Remarkably, 40 (32.6%) LT recipients had an underlying cholestatic disease.

Forty-six patients (37.4%) experienced EAD after LT. In 25 cases, a T-peak > 2000 IU/L was reported, followed by 10 cases with bilirubin ≥ 10 mg/dL and 7 cases with INR ≥ 1.6 at day seven after LT. In three patients, bilirubin ≥ 10 mg/dL and aminotransferase > 2000 IU/L were reported, while one recipient had all three EAD risk factors. Six (4.9%) patients experienced graft loss within 90 days.

The median values of the AST and ALT peaks after LT were 997 (IQR: 636–1718) IU/L and 706 (IQR: 447–1215) IU/L, respectively. The median values of INR and bilirubin on day 7 were 1.2 (IQR: 1.3–2.0) and 7.7 (IQR 1.5–4.3) mg/dL, respectively.

The 90-day patient and graft survival rates were 96.7% and 95.1%, respectively.

The median estGW (23) and estimated GRWR were 1359 (IQR: 1240–1442) g and 1.73 (IQR: 1.52–1.98), respectively. Thirty-seven (30.0%) recipients showed an estimated GRWR < 1.57; 67 (54.5%) between 1.57 and 2.13, and 19 (15.5%) > 2.13.

WIT and CIT were significantly lower in the Validation-Set cohort. All demographic and clinical characteristics of both the Training-Set and Validation-Set are described in Table 2.

**Table 2:** Main clinical characteristics of the Training-SET and Validation SET

Variables	Training-SET (*n*:331) Median (IQR) or Number (%)	Validation-SET (*n*:123)	*p* value
Recipient age at LT	57 (50–62)	55 (44–61)	0.051
Recipient bio MELD	15 (11–19) (Ranges: 6–37)	15 (9–23)* (Ranges: 6–40)	0.421
Recipient etiology liver disease			< 0.0001
✓ Autoimmune/cholestatic	7 (2.1)	40 (32.6)	
✓ No Autoimmune/cholestatic	324 (97.9)	83 (77.4)	
Recipient ICU stay	5 (3–8)	2 (2–3)	< 0.0001
Donor age	61 (42–70)	60 (49–69)	0.470
Donor gender (Male)	182 (55.0)	61 (49.6)	0.104
Donor AST (IU/L)	38 (24–74)	48 (24–52)	0.108
Donor bilirubin (mg/dl)	0.7 (0.5–1.0)	0.9 (0.5–1.3)	0.201
Donor sodium (peak value) mmol/L	151 (146–157)	149 (145–157)	0.332
Donor BSA (m^2^)	1.86 (1.75–1.98)	1.92 (1.75–2.03)	0.424
Macrovesicular steatosis > 30%	23 (6.9)	13 (10.6)	0.345
Graft weight (g)	1350 (1191–1648)	1359 (1240–1442)^†^	0.328
GRWR	1.83 (1.57–2.13)	1.73 (1.52–1.98)	0.111
WIT (min)	52 (45–56)	28 (24–33)	< 0.0001
CIT (min)	394 (350–440)	356 (300–420)	< 0.0001
LT time (min)	430 (390–460)	469 (416–527)	< 0.0001
EAD^2^	203 (61.3)	46 (37.4)	< 0.0001

*ICU* Intensive Care Unit, *AST* aspartate aminotransferase, *GRWR* graft to recipient weight ratio, *WIT* warm ischemic time, *CIT* cold ischemic time, *LT* Liver Transplantation, *BSA* body surface area, *EAD* early allograft dysfunction

*MELD score was calculated only for those recipients who underwent LT with no cholestatic disease indications; ^†^in the Validation-SET the graft weight and the GRWR were estimated using the following formula: estimated standard liver weight (g) = 218 + body weight (kg) × 12.3 + gender (female 0; male:1) × 51]

### Primary end-point

In multivariable logistic regression, four variables were identified as independent risk factors for 90-day graft loss. These included, all three components of EAD: a serum bilirubin ≥ 10 mg/dL on day 7 (OR = 2.04, 95%CI = 1.06–3.95; *p* = 0.03) an INR ≥ 1.6 on day 7 (OR = 2.25, 95%CI = 1.09–4.65; *p* = 0.03), and transaminase levels > 2000 IU/L within the first week after LT (OR = 2.24, 95%CI = 1.06–4.70; *p* = 0.03). In addition, GRWR was also identified, either at high values (> 2.13: OR = 2.56, 95%CI = 1.21–5.42; *p* = 0.01), or at low values (< 1.57: OR = 2.46, 95%CI = 1.11–5.44; *p* = 0.03), as reported in Table 3.

**Table 3:** Multivariable model for 90-day graft loss (modified-EAD)

Variables	Beta	OR	95% CI	*p* value
GRWR: 1.57–2.13	Ref	1.00	–	–
GRWR < 1.57	0.90	2.46	1.11–5.44	0.03
GRWR > 2.13	0.94	2.56	1.21–5.42	0.01
Total Bilirubin ≥ 10 mg/dl (on POD 7)	0.71	2.04	1.06–3.95	0.03
INR ≥ 1.6 (on POD 7)	0.81	2.25	1.09–4.65	0.03
AST > 2000 IU/L (within POD 7)	0.81	2.24	1.06–4.70	0.03
Constant	− 3.15	0.04	–	< 0.001

*GRWR* graft to recipient weight ratio, *POD* post-operative days, *AST* aspartate aminotransferase

Using these four variables, we constructed a new model, defined as the mEAD, based on the following formula:

$$ \begin{gathered} {2}.0{4 }\left( {{\text{if total bilirubin }} \ge {1}0{\text{ mg}}/{\text{dL}}} \right) \, \hfill \\ \quad + { 2}.{25 }\left( {{\text{if INR }} \ge {1}.{6}} \right) \hfill \\ \quad + { 2}.{24 }\left( {{\text{if AST}} \ge {2}000{\text{ IU}}/{\text{L}}} \right) \hfill \\ \quad + { 2}.{56 }\left( {{\text{if GRWR}} > {2}.{13}} \right) \hfill \\ \quad + { 2}.{46 }\left( {{\text{if GRWR }} < {1}.{57}} \right) \, {-}{ 3}.{15} \hfill \\ \end{gathered} $$

**Fig. 1:**
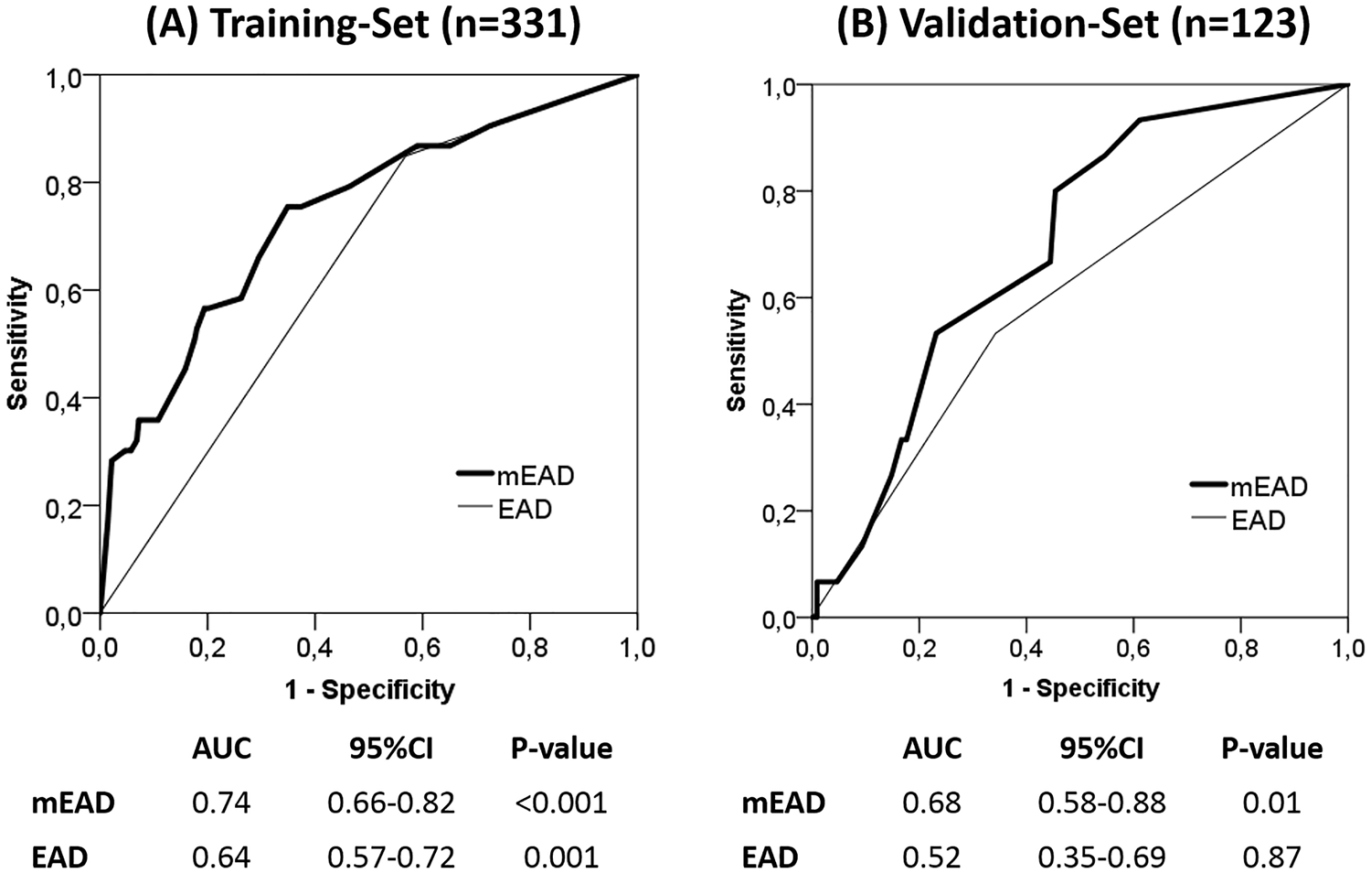
Evaluation and comparison of the m-EAD and EAD predicting models on 90-day graft loss [Training-SET (**A**) and Validation-SET (**B**)]

The new mEAD model showed better predictive capability for 90-day graft loss than the conventional EAD model^1^ in the Training-Set (AUC = 0.74, 95%CI = 0.66–0.82; *p* < 0.001 vs. AUC = 0.64, 95%CI = 0.57–0.72, *p* = 0.001) and in the Validation-Set (AUC = 0.68, 95%CI = 0.58–0.88; *p* = 0.01 vs*.* AUC = 0.52, 95%CI = 0.35–0.69; *p* = 0.87) (Fig. 1).

In the Training-Set, the mEAD model showed an NRI of 0.93 vs. the EAD, resulting from a correct reclassification of 0.4% of events (graft loss) and 8.9% of non-events (no-graft loss). Similarly, in the Validation Set, we observed a positive NRI value of 0.23 due to a correct reclassification of 0.5% of events and 1.8% of non-events. In the Training Set, the adopted *z*-test showed a significantly superior diagnostic ability of the mEAD vs. EAD (*z* = 2.76, *p* = 0.006). Although the mEAD was superior in terms of diagnostic ability (*z* = 1.54) in the Validation Set, the test only merged statistical significance (*p* = 0.12).

**Fig. 2:**
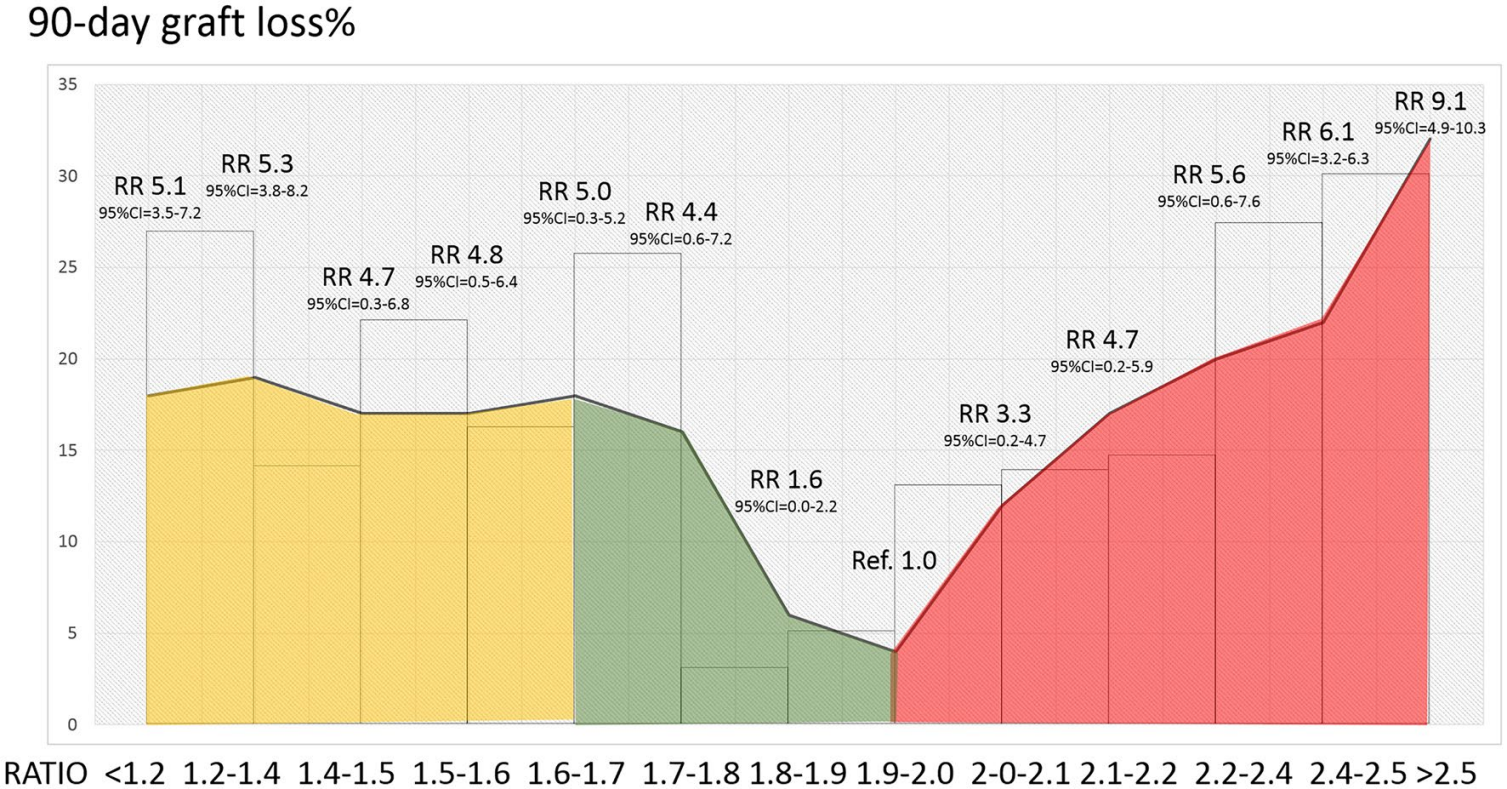
Relative risk of 90-day graft loss according to the GRWR. The histograms represent the Training-Set population distribution according to the GRWR (reported on the right *y*-axis). The left *y*-axis shows the 90-day graft loss incidence (%). The line represents the relative risk (RR) of 90-day graft loss according to the GRWR. The GRWR RR = 1 represents the reference (median GRWR value = 1.9); the “red zone” represents the increase in the RR of 90-day graft loss when the GRWR > 2.0. Contrarily, the “green zone” represents the decrease in the risk (from GRWR = 1.57 to GRWR = 2.0). The RR curve remains stable (RR $\sim$ 5) when GRWR < 1.6 (yellow zone)

**Fig. 3:**
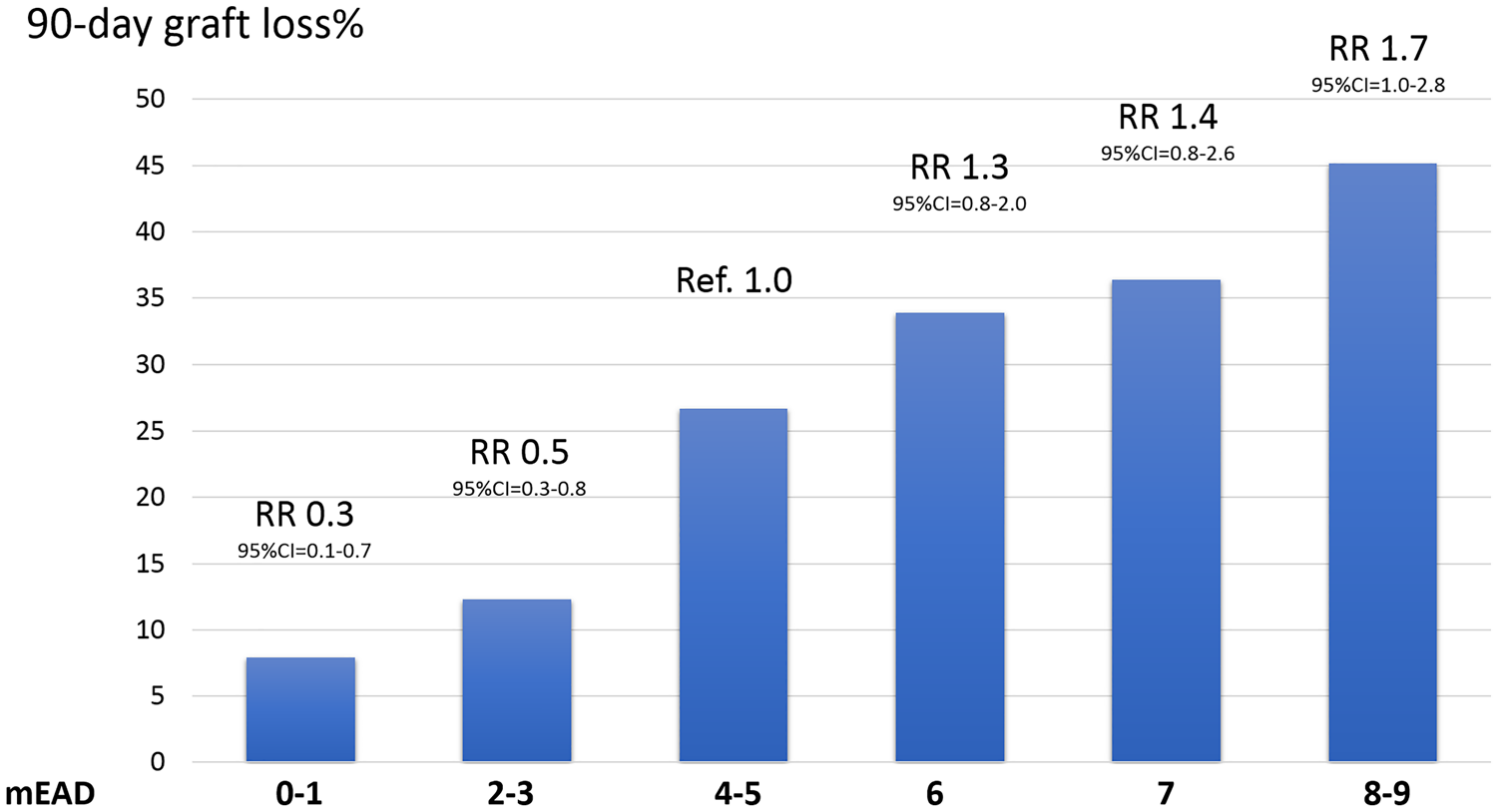
Relative risk of 90-day graft loss according to the mEAD. The histograms represent the Training-Set population distribution according to the mEAD (reported on the right *y*-axis). The left *y*-axis shows the 90-day graft loss incidence (%). The line represents the relative risk (RR) of 90-day graft loss according to the mEAD. The mEAD RR = 1 represents the reference. (median mEAD value = 4.3)

The relative risk (RR) for 90-day graft loss according to the GRWR and mEAD are depicted in Figs. 2 and 3. The GRWR and mEAD values between 1.9–2.0 and 4–5, respectively, were considered reference points. These cut-offs were selected because they corresponded to the Training Set's median GRWR and mEAD values. High RR was observed in both the extremes of the GRWR curve and for upper mEAD values, respectively. For example, a RR for 90-day graft loss of 5.1 (95%CI = 3.5–7.2) was reported if the GRWR was < 1.2. A GRWR ranging from 1.2 to 1.4 was associated with a RR of 5.3 (95%CI = 3.8–8.2). Similarly, a RR = 6.1 (95%CI = 3.2–6.3) was observed when the GRWR ranged between 2.4 and 2.5, and RR = 9.1 (95%CI = 4.9–10.3) if the GRWR was $\ge$ 2.5.

In the case of mEAD classes of risk, patients with a mEAD value of 0–1 (RR = 0.3) and 2–3 (RR = 0.5) had low relative risks for 90-day graft loss when compared with patients with a mEAD value of 4–5 (considered as a reference value). On the opposite, increased values corresponded to an increased RR (score 6: RR = 1.3; score 7: RR = 1.4; score 8–9: RR = 1.7).

### Secondary end-point

The median T-peak observed within the first week after LT was 974 (IQR: 530–1830) IU/L. Sixty-eight (20.5%) LT recipients experienced a T-peak > 2000 IU/L. The median GW in recipients presenting with a T-peak $>$ 2,000 IU/L vs*.* those with a T-peak < 2000 IU/L were 1500 g (IQR: 1365–1748) and 1306 g (IQR: 1156–1540) (*p* < 0.0001), respectively. Using multivariate analysis, we investigated the effect of GW on the risk of presenting with a T-peak $>$ 2000 IU/L after adjustment for donor age, donor ICU-stay, WIT, CIT, steatosis grade, and MELD score. As shown in Table 4, GW was found to be an independent risk factor for T-peak $>$ 2000 IU/L (OR = 2.385 per 500 g of GW, 95%CI 1.469–3.925, *p* = 0.0005). Among the other considered potential risk factors, the steatosis grade was the only one that showed a significant risk, with an estimated OR of 1.43 for every 10% increase in steatosis.

**Table 4:** Multivariable logistic regression model for the transaminase > 2000 IU within the first post LT week

Variables	Beta	OR	95% CI	*p* value
Graft weight (g)	1.74	2.39*	1.47–3.93	0.0005
Macrovesicular steatosis (%)	0.04	1.04	1.01–1.07	0.01
WIT (min)	− 0.02	0.98	0.96–1.002	0.09
CIT (min)	0.002	1.002	0.999–1.01	0.18
Donor ICU stay (min)	0.02	1.02	0.98–1.06	0.34
Donor age (years)	− 0.002	0.998	0.98–1.02	0.78
MELD	− 0.04	0.96	0.92–1.01	0.10
Constant	− 3.16			0.01

*WIT* warm ischemic time, *CIT* cold ischemic time, *ICU* Intensive Care Unit, *BMI* body mass index

*OR was calculated per 500 gr GW increasing. An increasing in GW of 100 gr correspond to a 19% rising in recipients who experience T-peak > 2000 (OR: 1.190; CI = 1.080–1.315, *p* = 0.0005)

## Discussion

Historically, in adult patients who have undergone LT, the EAD model proposed by Olthoff et al. in 2010 [1] has been a reasonably good discriminator of the probability of early graft loss. Both allograft dysfunction and loss are ultimately influenced by donor-related factors such as the donor age [16] and the use of extended donor criteria (ECD) [4, 5]. So far, attempts to optimize matching between the donor graft and the recipient have been primarily pursued by taking into account the donor risk criteria [17] and the clinical status of the recipients based on individual MELD scores. In adults, the donor-recipient size match has rarely been taken into consideration, as in most countries (either in the Eurotransplant zone or the US), the allocation system is essentially based on the principle of “the sickest first,” which implies that any given graft is assigned depending on liver disease severity, with little regard to donor and recipient morphologies [18].

In Italy, the organ allocation system is based on the concept of urgency, utility, and “transplant benefit” [19], as regulated by the national transplant network policy [20]. Therefore, with the sole exception of recipients classified as United Network for Organ Sharing (UNOS) Status 1 and those with a MELD score > 29 (who deserve national or macro-area sharing), all grafts are shared at a regional level, and each transplant center may choose the best recipient based not only on urgency but also on the recipient and donor-size matching.

The GRWR represents a highly sensitive and critical parameter in pediatric patients [21]. Even though in a few centers, a GRWR up to 4 has been considered for LT, a GRWR > 2.5 is inadvisable in both pediatrics [22] and adult patients [18], since the “large-for-size syndrome” might be responsible for morbidities and vascular complications. Although assessing an appropriate GRWR is a crucial issue during the allocation procedure in the pediatric population, this parameter has been poorly investigated [18] in the adult LT setting, where it uncommonly represents a reason to decline a donor or select another recipient from the waiting list. The French authors reported that GRWR $\ge$ 2.5 did not influence graft and patient outcomes [18]. In contrast, our study suggests that the GRWR plays an essential role in determining 90-day outcomes. The dissimilarities between our and the French experiences could be explained by the differences in the study aims and the explored GRWR ranges. The French authors mainly investigated the impact of GRWR $\ge$ 2.5 on early postoperative morbidity and outcomes; meanwhile, our paper was primarily focused on the role of lower GRWR ranges (IQR: 1.57–2.13; GRWR > 2.5 being observed in only 8% of our cohort) on early graft outcomes. The French study was designed to assess the significance of using large grafts on small recipients. Conversely, we wanted to construct a multifactorial model to address the impact of “*GRWR variability*” on 90-day graft survival. Since LT early outcome models taking into account preoperative donor/recipients variables as well as intraoperative features (i.e., CIT and WIT) have been reported so far [23, 24], in order to predict 90-day graft survival, we decided to propose a new formula which included the *perioperative* donor-recipient size match (i.e., GRWR) in association with the well-known early *postoperative* liver functions variables (i.e., PT-INR, bilirubin, transaminase peak).

The newly proposed mEAD model, which is an adjustment of the conventional Olthoff EAD model, showed that both high ($>$ 2.13) and low ($<$ 1.57) GRWR values are significant predictors of 90-day graft loss. Indeed, the mEAD-model performed better than the conventional EAD-model to predict 90-days graft loss, showing a significant increase in c-statistics. The strength of the mEAD predictive model compared with the conventional EAD applied in the training cohort was also confirmed after external validation. Unfortunately, although the mEAD was superior also in the external validation in terms of diagnostic ability (*z* = 1.54), the test only merged statistical significance when compared with the Olthoff EAD (*p* = 0.12). This datum underlines the relative impact of mEAD when compared with the original EAD in the external validation. However, the fact that the p-value was 0.12 does not mean that no effect exists. The AUC of mEAD was superior (0.68 vs. 0.52), consenting to reclassify a relevant number of patients correctly. In detail, the mEAD model correctly reclassified 0.4% of graft loss and 8.9% of no-graft loss (NRI = 0.93) in the Training-Set and 0.5% of graft loss and 1.8% of no-graft loss in the Validation-Set (NRI = 0.23). The small number of cases (*n* = 123) and events of interest (only 4.9% of 90-day graft losses) reported in training set strongly conditioned the results of c-statistics. The smaller the number of events of interest, the greater the AUC confidence intervals (ranging 0.35–0.69), the greater the overlapping of the AUC values between mEAD and EAD, and inferior the statistical significance observed comparing the tests.

Even if the newest scores as EASE (AUC = 0.87, 95%CI 83–91%) [25] and L-Graft (AUC = 0.78, 95%CI 0.75–0.82) [26] outperformed the EAD, we decided to build the model considering the binary Olthoff’s variables because (i) to date, the Olthoff’s model is the most cited and widely used definition of EAD [27], (ii) our data analysis was conducted before the validation of the L-Graft and EASE score, and (iii) both the L-Graft and EASE score are mathematically complicated, and its results could be not generalizable in different transplantation environments.

As shown in Fig. 2, the risk of 90-day graft loss decreases slowly beyond a GRWR value > 1.6, and then it increases again when it exceeds 2.0. Hence, our data suggest that the best donor-recipient match is achieved with a GRWR value of 1.6–2.0, a range that can be considered as a “safety window.” Concerning the transplant benefit principle [19], we suggest considering the GRWR during the adult organ allocation process. The GW should always be estimated before organ acceptance to make this possible. Ultrasonography, an operator-dependent technique, may be capable of estimating the extent of steatosis and the dimensions of the liver but cannot precisely measure the GW. The computed tomography-based liver volumetry scan could be a much better option for this purpose, but it is expensive and not always readily available at many hospitals offering donors. Practically, estimation of the GW by currently available formulas [10, 28] may represent the easiest option to obtain an approximation of the GRWR prior to allocation of the liver. The weight and height of the donor and the BSA The ratio between the GW and the right anteroposterior distance between the recipient's ribs could be a further option to find out the best donor and recipient morphological match [29].

Our study also shows that GW allowed estimation of the level of the post-transplant T-peak. This finding may be a consequence of the increased difficulty in liver perfusion of large and heavy grafts during procurement and slower reperfusion at LT. Undoubtedly, the homogeneous intrahepatic distribution of the perfusion solution during organ procurement or blood during reperfusion is influenced by the organ's size. Furthermore, GW may be influenced by the extent of macrosteatosis, leading to partial or complete sinusoid obstruction [30], the Kupffer cell dysfunction, or an increase in leukocyte adhesion and lipid peroxidation [7], all of which are associated with hepatocellular death and the T-peak.

We are aware that the high incidence of the EAD [1, 2] observed in our training cohort may represent a potential limitation. This was mainly related to only one EAD component above the given threshold, reflecting a high frequency of unfavorable donor-related features. About 40% of deceased donors were older than 65 years, 8% had > 30% macrovesicular steatosis, and 7% had a sodium level > 160 mmol/L. Reasonably, the high incidence of EAD could also be justified by the fact that: (i) all ECD livers which were enrolled in the present study were not reconditioned or tested for viability with hypothermic or normothermic machine perfusion, respectively [31, 32] not yet available at Training-Set Centres at time of the enrollment) (ii) the CIT was significantly higher in the Training-Set (*p* < 0.0001) (due to the difference in the surface area and transplant logistics in the two countries) (iii) an extended WIT could also justify the higher EAD incidence in the Training-Set cohort. Since a high incidence of the EAD can hamper the outcome of the primary end-point of the study, the mEAD model was externally validated. The study has other potential limitations that cannot be ruled out: (i) like other models mentioned above [20, 21], the mEAD is a “hybrid formula” mixing preoperative and early postoperative variables and should therefore be applied considering this limitation; (ii) the lower limit of 95%CI *c*-statistics in the Validation-Set is close to 0.50; (iii) it is multicentred and retrospective, with a limited number of transplanted patients, and (iv) since the GW in the Validation-Set cohort was not available at backbench, the GRWR were calculated using the estimation standard liver weight formula; hence we cannot exclude the possibility of minor inaccuracies in estimating GRWR. Finally, GRWR values ranged between 1.0 and 2.13 in 75% of LTs; thus, we cannot make firm conclusions in the case of a higher GRWR commonly used in other Western countries [18, 33].

In conclusion, this study suggests that, in the setting of whole adult LT, surgeons should pay greater attention to the measurement of GW, which should always be obtained at the backbench or estimated using the appropriate formulas [10, 28]. The newly proposed mEAD model, which incorporates the GRWR into the conventional EAD equation, allows an improved prediction of 90-day graft loss. Hence the GRWR may be considered during the graft allocation process.

## Data Availability

The datasets used during this study are available on reasonable request from the corresponding author.
